# Nutricosmetic effects of *Asparagus officinalis*: a potent matrix metalloproteinase-1 inhibitor

**DOI:** 10.1038/s41598-021-88340-2

**Published:** 2021-04-22

**Authors:** Suwannee Sriyab, Nachtharinee Laosirisathian, Chanun Punyoyai, Songyot Anuchapreeda, Singkome Tima, Sawitree Chiampanichayakul, Wantida Chaiyana

**Affiliations:** 1 Department of Pharmaceutical Sciences, Faculty of Pharmacy, Chiang Mai University, Chiang Mai, 50200 Thailand; 2 Research Center of Pharmaceutical Nanotechnology, Chiang Mai University, Chiang Mai, 50200 Thailand; 3 Division of Clinical Microscopy, Department of Medical Technology, Faculty of Associated Medical Sciences, Chiang Mai University, Chiang Mai, 50200 Thailand

**Keywords:** Biochemistry, Chemical biology

## Abstract

This study aimed to investigate the nutricosmetic effect of *Asparagus officinalis* extracts. The tip and spear of *A. officinalis* were successively extracted with 95% ethanol. The rutin, phenolic, and flavonoid contents of *A. officinalis* extracts were investigated. The antioxidant activities were determined by 2,2-azinobis (3-ethylbenzothiazoline-6-sulphonic acid) and a ferric reducing antioxidant power assay. Matrix metalloproteinase-1 (MMP-1), elastase, and hyaluronidase inhibition were determined by in vitro enzyme reaction assay. The cytotoxicity was analyzed on peripheral blood mononuclear cellss. Findings revealed that drying temperature and drying duration had significant effects on the chemical composition and biological activity of *A. officinalis* extract. *A. officinalis* tips dried at 50 °C for 24 h contained the (significantly) highest flavonoid and rutin content. The most potent extract was from *A. officinalis* spears since it possessed the (significantly) highest MMP-1, elastase, and hyaluronidase inhibition rates of 83.4 ± 1.5%, 70.4 ± 4.1%, and 75.2 ± 1.0%, respectively. Interestingly, at the same concentration, the *A. officinalis* spear extract was more potent in MMP-1 inhibition than oleanolic acid and epigallocatechin gallate, the well-known natural MMP-1 inhibitors. The results show that *A. officinalis* extract is an attractive source of natural anti-skin-wrinkle ingredients.

## Introduction

*Asparagus officinalis*, internationally known as “the king of vegetables”, is a perennial herb with various nutritional and bioactive components^1^. It originated from the eastern Mediterranean and Asia Minor, but at present it is widely cultivated in more than 60 countries around the world^1^. *A. officinalis* stalks are commonly eaten as a vegetable and have become one of the world’s top ten dishes due to its high nutritional value^1,2^. However, only one-fourth of the *A. officinalis* plant is eaten since the remaining three-fourths are discarded as by-products in the form of hard stems, roots, or leaves^3^. Additionally, mixing disparate sizes of *A. officinalis* stalks could lead to lower quality and cheaper selling price. Therefore, *A. officinalis* stalks that are smaller in size are excluded and separately sold at a lower price. Although these by-products and lower grade of *A. officinalis* are reported to contain similar phytochemicals^3^, their commercial value is not high. In some cases, they were processed into food products, including canned products, drinks, tea, wine, vinegar, yogurt, noodles, etc.^1^.

In recent years, *A. officinalis* has been widely used for medicinal purposes because it is rich in amino acids, folic acid, ascorbic acid, phenols, saponins, dietary fiber (non-starch polysaccharides), anthocyanins, etc.^4–7^ The effects of drying methods (e.g., vacuum, far-infrared, spouted bed, tray, and freeze drying) have been previously reported to affect the structural characterization, color quality, rehydration capacity, bioactive substances, and biological activities of *A. officinalis*^8–10^. Therefore, suitable drying conditions crucially affect the biological and commercial value of *A. officinalis. A. officinalis* has been linked with several beneficial biological activities and has potential use for anti-cancer, anti-tumor, immunomodulatory, hypoglycemic, anti-hypertensive, and anti-epileptic effects^8,11^. However, there are very few nutricosmetic applications of *A. officinalis.* Aqueous extract of *A. officinalis* has been reported as a natural inhibitor against tyrosinase activity and browning reaction^12^. The mechanism of melanin inhibition in the skin encompasses the reduction of intermediates and further prevention of o-quinone transformation to melanin^13^. Therefore, *A. officinalis* has potential use as a natural whitening compound. However, the anti-wrinkle activities and beneficial effects of *A. officinalis* extract on skin ageing have not been reported before. Currently, anti-wrinkle products are of interest worldwide due to the ageing population, which has led to a significant increase in the number of elderlies.

Therefore, this study is the first to report on the nutricosmetic effect of *A. officinalis* extract in relation to matrix metalloproteinase-1 inhibition. Moreover, the chemical compositions and antioxidant activities of *A. officinalis* extract were determined. Since the drying process is reported to influence both the chemical composition and biological activity of *A. officinalis*^8^, the effects of drying temperature and drying duration on the chemical composition and nutricosmetic activity were also investigated.

## Results and discussion

### *A. officinalis* extracts

Three factors affecting the yield, chemical composition, and nutricosmetic effect of *A. officinalis* were investigated, including *A. officinalis* parts used (spear or tip), drying temperature (50 °C or 80 °C), and drying duration (24 or 32 h). Drying duration was the first factor investigated. The spear part of *A. officinalis* was selected as the plant material, and the drying temperature was set to 50 °C. The results showed that a drying duration longer than 24 h was unnecessary since the *A. officinalis* material was completely dried at 24 h. Therefore, the suggested drying duration for further experiments was 24 h. *A. officinalis* dried in various conditions had different external appearances. Higher drying temperature and longer drying duration led to a remarkable darker brown color. However, all *A. officinalis* ethanolic extracts exhibited the same external appearance, which was a dark green, semisolid mass with characteristic odor. The yields of each extract were in the range of 6.6% w/w to 13.0% w/w, as shown in Table 1.

**Table 1:** *A. officinalis* extracts and their raw materials.

*A. officinalis *parts used	Drying temperature (°C)	Drying duration (h)	Yield (% w/w)
Spear	50	32	9.8
	50	24	13.0
	80	24	11.4
Tip	50	24	6.6
	80	24	10.5

### Chemical composition of *A. officinalis* extracts

**Figure 1:**
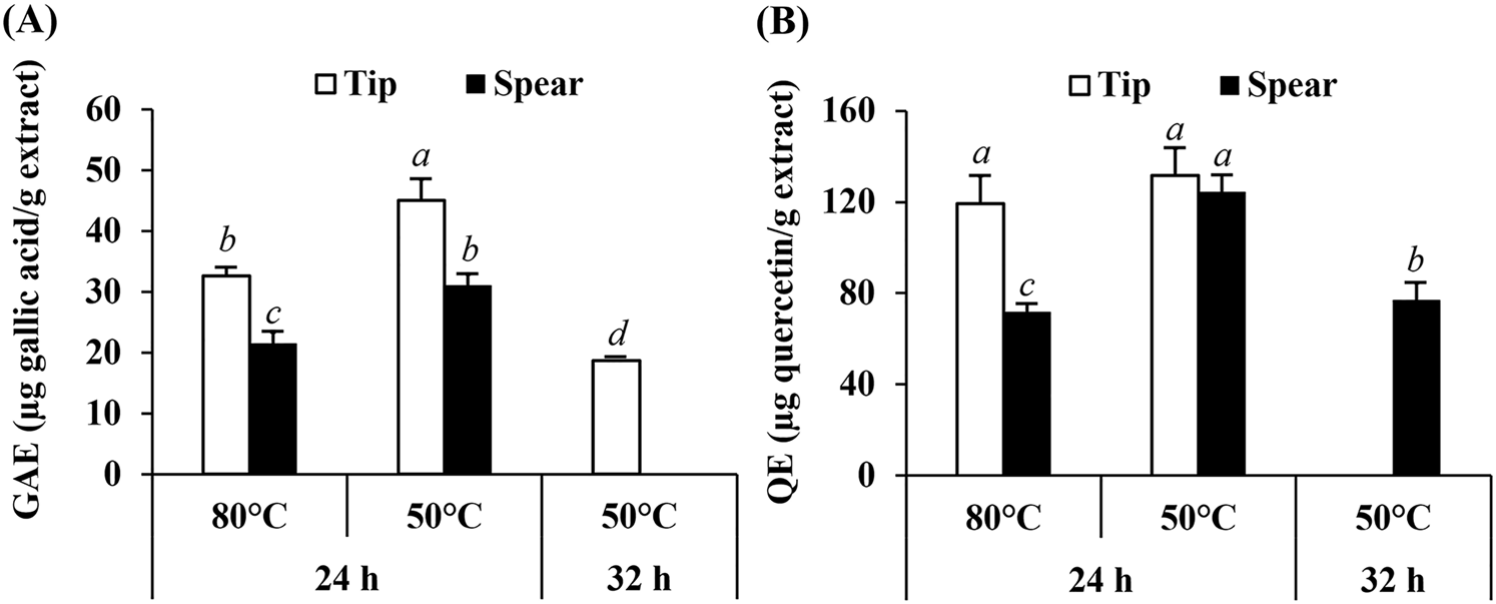
Total phenolic content in terms of gallic acid equivalent (GAE) of *A. officinalis* extracts prepared at different drying temperatures and drying duration (**A**). Total flavonoid content in terms of quercetin equivalent (QE) of *A. officinalis* extracts prepared at different drying temperatures and drying duration (**B**). Different letters, a, b, c, and d, denote significant differences in phenolic or flavonoid content among various *A. officinalis* extracts, whereas similar letters denote non-significant differences at *p* < 0.05.

The total phenolic and total flavonoid content are expressed as gallic acid equivalent (GAE) and quercetin equivalent (QE), respectively. The GAE and QE of *A. officinalis* ethanolic extracts are shown in Fig. 1. The present study found that *A. officinalis* ethanolic extracts were rich in flavonoids. Both tip and spear parts contained non-significantly different flavonoid content of 131.8 ± 11.7 and 124.3 ± 7.6 mg quercetin per g extract (Fig. 1B), respectively, whereas, *A. officinalis* tips contained significantly higher phenolic content than the spear (Fig. 1A) (*p* < 0.05). *A. officinalis* tips dried at 80 °C for 24 h contained the (significantly) highest phenolic content of 45.1 ± 3.5 μg gallic acid/g extract (Fig. 1A). This might be due to the reduction in water activity during the drying process that led to the retardation of chemical reactions and, hence, preserved the chemical and bioactive nutrients^14^. A previous study reported that the phenolic contents of *Lycopersicon esculentum* L. and *Capsicum annum* were best preserved after drying at 60 °C, compared to 40 °C and 50 °C^14^.

On the other hand, drying temperature had no effect on the total flavonoid content of *A. officinalis* tips. Although the flavonoid content in spear extracts was lower at 80 °C than at 50 °C, in the tip extracts the content was equivalent between 80 and 50 °C (Fig. 1B). A likely explanation might be due to the more delicate structure of the *A. officinalis* tip compared to the spear, for which the temperature affected their chemical compositions. Therefore, the flavonoids could be degraded at only 50 °C, and the total flavonoid content of the *A. officinalis* tip dried at 80 °C and 50 °C was not significantly different. Conversely, due to the tougher structure of the spear, only some flavonoids were degraded at 50 °C. A higher temperature (80 °C) was needed to degrade the flavonoids inside the *A. officinalis* spear. Hence, flavonoid content in the spear extracts was lower at 80 °C than at 50 °C.

In contrast, a higher drying temperature led to significantly higher phenolic content but lower total flavonoid content (Fig. 1) (*p* < 0.05). Therefore, it might be assumed that phenolic compounds are more stable than flavonoids at high temperature. These findings were well supported by Lu et al.^15^, who reported that the nutritional value of A. officinalis decreased significantly as a function of blanching time and temperature. At blanching temperatures below 70 °C, the total phenolic content slightly decreased by 3.93% and 14.25% after 80 and 160 min, whereas total flavonoid content remarkably reduced by 13.34% and 23.02% after blanching for 80 and 160 min, respectively^15^. Additionally, a previous study reported that thermal processing caused a significant reduction in total soluble phenolic content but an increase in total insoluble-bound phenolics^16^. Therefore, alteration of phenolic compounds after drying in a hot-air oven depended on the type of phenolic compound.

In addition to drying temperature, the drying duration also had an effect on both phenolic and flavonoid content of *A. officinalis* ethanolic extracts (Fig. 1). A longer drying duration led to significantly lower phenolic and flavonoid content (*p* < 0.05). A likely explanation is due to the degradation of some phenolics and flavonoids during the drying process^15,16^. Drying methods, especially hot air drying, had great effect on phenolic content^17^.

**Figure 2:**
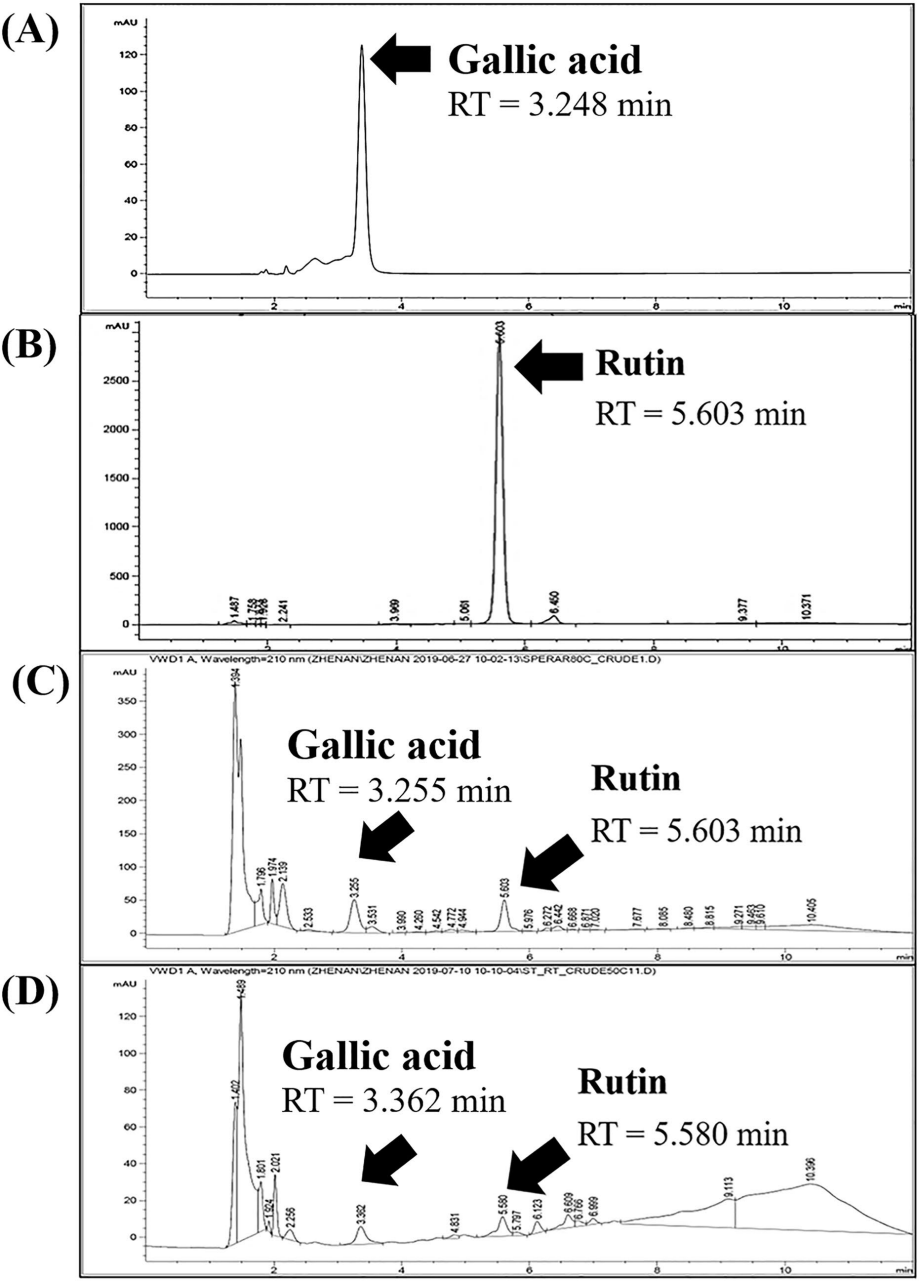
HPLC chromatograms of standard gallic acid (**A**), standard rutin (**B**), *A. officinalis* tip extracts (50 °C, 24 h) (**C**), and *A. officinalis* spear extracts (50 °C, 24 h) (**D**).

Since rutin was reported as the main flavonoid present in the tip and spear of *A. officinalis*^18^, the content of rutin was also investigated in the present study. HPLC chromatograms of standard gallic acid, standard rutin, and *A. officinalis* extracts are shown in Fig. 2. There were several peaks detected in the extracts of *A. officinalis.* The main peaks detected at the retention times of 3.255 and 3.362 min in HPLC chromatograms of *A. officinalis* tip extracts (50 °C, 24 h) and spear extracts (50 °C, 24 h), respectively. These peaks correlated well with the peak of standard gallic acid (retention time = 3.248 min). Therefore, gallic acid was remarked as one of the main components in both *A. officinalis* tip and spear extracts. On the other hand, there are also the peak at 5.603 and 5.580 min with similar peak area and peak height that could be also the main component. The retention times of these peaks correlated well with the peak of standard rutin (retention time = 5.603 min). Therefore, both gallic acid and rutin were remarked as the main components in both *A. officinalis* tip and spear extracts.

**Figure 3:**
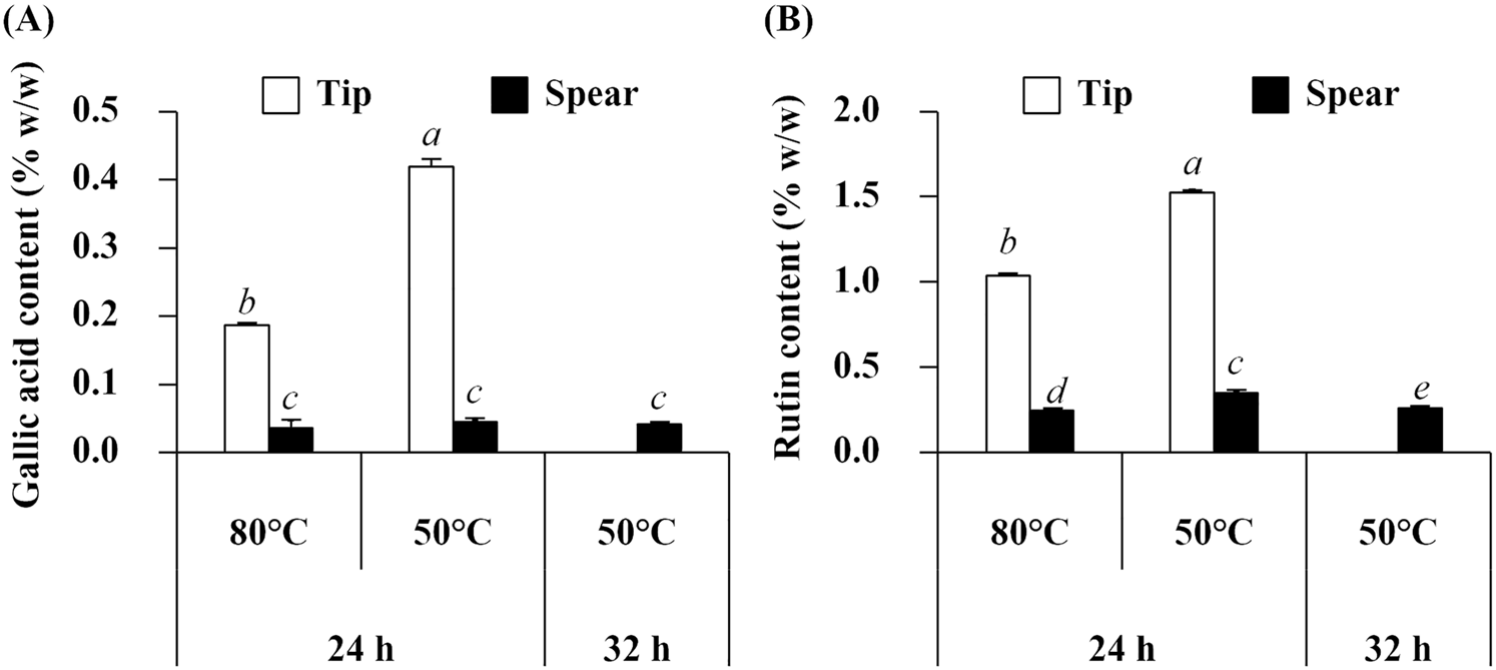
Gallic acid content (**A**) and rutin content (**B**) of *A. officinalis* extracts prepared at different drying temperatures and drying duration. Different letters, a, b, c, d, and e, denote significant differences in rutin content among various *A. officinalis* extracts, whereas similar letters denote non-significant differences at *p* < 0.05.

The gallic acid and rutin content of *A. officinalis* extracts showed a similar pattern (Fig. 3). *A. officinalis* tips contained a significantly higher gallic acid and rutin content than the spear (*p* < 0.05). The results were in accordance with previous studies reporting that the *A. officinalis* tip was a rich source of gallic acid and rutin^18,19^. In addition, the present study revealed that both drying temperature and drying duration significantly affected the gallic acid and rutin content in both *A. officinalis* tip and spear. However, gallic acid contents were not found to be significantly different in *A. officinalis* spear drying under different conditions. The (significantly) highest gallic acid (0.42 ± 0.01% w/w) and rutin (1.52 ± 0.02% w/w) content was detected in an extract from the *A. officinalis* tip, which was dried at 50 °C for 24 h.

In addition to gallic acid and rutin, *A. officinalis* has been reported to contain several chemical constituents, such as asparagusic acid, ketone vanillin, thiazole, thiophene, and their methyl and ethyl esters^20^. The combination of these chemical constituents could lead to the synergistic effects and confer superior biological activity to *A. officinalis*. Although other vegetables also contained some similar chemical components, lower level of these compounds would result in a higher dose requirement. *A. officinalis* has been reported to contain higher flavonoids level and possess higher antioxidant than *Brassica oleracea*^21^*,* while some mineral elements, especially organic selenium, of *A. officinalis* were much higher in *A. officinalis* than in other vegetables, pork, eggs, and even mushrooms^22^. Although there was some evidence that *A. officinalis* contains an abundance of biologically active compounds and is referred to as the "king of vegetables"^22^, the present study investigated its antioxidant and anti-wrinkle properties to confirm its potential use in the nutraceutical and/or cosmeceutical industry.

### Antioxidant activitiess of *A. officinalis* extracts

Antioxidant activities of *A. officinalis* extracts are shown in Table 2. Since at least two different test methods were suggested to determine the antioxidant activity, the ABTS assay (radical scavenging or antiradical activity) and FRAP assay (reducing capacity of ferric ions in the compounds) were performed in the present study^23,24^. The results showed that the extract from *A. officinalis* tips, which were dried at 50 °C for 24 h, was the most potent antioxidant, with the (significantly) highest EC_1_ value of 9.6 ± 0.8 μM FeSO_4_/g extract and the (significantly) highest TEAC value of 32.6 ± 2.1 mg Trolox/g extract. The antioxidant activities were related to their chemical composition profile since the extract from *A. officinalis* tips that contained the highest amount of phenolic, flavonoids, and rutin possessed the (significantly) highest antioxidant activity.

**Table 2:** Antioxidant activities of *A. officinalis* extracts.

Sample	Drying temperature (°C)	Drying duration (h)	EC_1_ (μM FeSO_4_/g extract)	TEAC (mg Trolox/g extract)
Ascorbic acid	–	–	238.2 ± 2.4^b^	124.0 ± 0.4^a^
Gallic acid	–	–	244.4 ± 0.9^a^	122.6 ± 0.6^b^
Quercetin	–	–	154.8 ± 1.9^c^	122.7 ± 0.4^b^
Rutin	–	–	106.6 ± 2.2^d^	120.9 ± 0.5^c^
*A. officinalis* spear	50	32	1.8 ± 0.5^h^	4.1 ± 1.0^g^
	50	24	3.4 ± 0.0^g^	13.0 ± 1.3^f^
	80	24	1.8 ± 0.2^h^	11.2 ± 3.6^f^
*A. officinalis* tip	50	24	9.6 ± 0.8^e^	32.6 ± 2.1^d^
	80	24	7.5 ± 0.3^f^	21.6 ± 1.3^e^

Data presented as mean ± SD (n = 3).

Different letters, a, b, c, d, e, f, g, and h, denote significant differences in antioxidant activities among various *A. officinalis* extracts, whereas similar letters denote non-significant differences at *p* < 0.05.

Gallic acid had the most potent radical scavenging and reducing capacity. Therefore, the antioxidant activities should be related to the total phenolic content. However, *A. officinalis* tips dried at 80 °C for 24 h, which contained the significantly highest total phenolic content, did not possess the highest antioxidant activity (i.e., radical scavenging and reducing capacity). Hence, it could be summarized that gallic acid was not the major phenolic component of *A. officinalis.* Apart from gallic acid, there are other abundant phenolic compounds in *A. officinalis* such as 3-O-feruloylquinic acid, asparanin, asparoffin, asparenyol, gobicusin, etc.^25,26^ Therefore, although the phenolic content in spear and tip extracts was higher at 80 °C than at 50 °C (Fig. 1A), the antioxidant activities in spear and tip extracts were lower at 80 °C than at 50 °C (Table 2).

In contrast, the antioxidant activity of *A. officinalis* extracts was related to their flavonoid content. Both flavonoids in the present study, including quercetin and rutin, exhibited strong reducing capacity, which was comparable to that of gallic acid and ascorbic acid. Therefore, both quercetin and rutin are suggested as the biological compounds responsible for the antioxidant activities of *A. officinalis* extracts.

On the other hand, both drying temperature and drying duration affected the antioxidant activity of *A. officinalis* extracts. A higher drying temperature and longer drying duration led to lower antioxidant activities via both radical scavenging and reducing capacity. Flavonoids, including rutin and quercetin, were suggested as the compounds responsible for the different activities between 80 and 50 °C since the antioxidant activity of *A. officinalis* extracts related well with the content of flavonoids (Fig. 1) and rutin (Fig. 3). However, it was noted that the antioxidant activities of *A. officinalis* ethanolic extracts were not potent enough since they were much lower than that of ascorbic acid, a well-known natural antioxidant compound.

### Anti-wrinkle activities of *A. officinalis* extracts

Anti-wrinkle activities of *A. officinalis* extracts are shown in Table 3. Quercetin was noted as a major component in *A. officinalis* extracts, which possessed the most potent inhibitory activities against MMP-1 and elastase, whereas gallic acid was the most potent hyaluronidase inhibitor. However, the anti-wrinkle activities of these pure compounds were not as potent as the positive control (oleanolic acid and EGCG), except for MMP-1 inhibition of quercetin, which was comparable to that of EGCG.

**Table 3:** Inhibitory activities of *A. officinalis* extracts on MMP-1, elastase, and hyaluronidase.

Sample	Drying temperature (°C)	Drying duration (h)	MMP-1 inhibition (%)	Elastase inhibition (%)	Hyaluronidase inhibition (%)
Oleanolic acid	–	–	71.7 ± 0.1^b^	–	–
EGCG	–	–	58.5 ± 6.9^c^	89.6 ± 3.0^a^	86.0 ± 1.1^a^
Gallic acid	–	–	7.3 ± 1.8^e^	18.8 ± 1.5f.	71.9 ± 1.2^c^
Quercetin	–	–	54.3 ± 8.7^c^	66.4 ± 2.6^b^	49.7 ± 1.7^e^
Rutin	–	–	23.5 ± 3.3^d^	7.3 ± 2.9^ g^	58.4 ± 1.5^d^
*A. officinalis* spear	50	32	70.9 ± 0.5^b^	57.3 ± 3.0^c^	58.2 ± 1.8^d^
	50	24	83.4 ± 1.5^a^	70.4 ± 4.1^b^	75.2 ± 1.0^b^
	80	24	59.9 ± 2.4^c^	41.2 ± 4.0^e^	70.9 ± 2.2^c^
*A. officinalis* tip	50	24	70.2 ± 2.9^b^	47.1 ± 1.7^d^	79.1 ± 0.7^b^
	80	24	57.3 ± 0.7^c^	40.7 ± 0.6^e^	73.9 ± 2.0^c^

The concentration of *A. officinalis* extracts used in the test was 0.1 mg/ml. Data presented as mean ± SD (n = 3)

Different letters, a, b, c, d, e, f, and g. denote significant differences in antioxidant activities among various *A. officinalis* extracts, whereas similar letters denote non-significant differences at *p* < 0.05.

Although the contents of phenolics, flavonoids, and rutin are higher in tip extract than in the spear extract at 50 °C, these compounds did not play an important role in the anti-wrinkle activities of *A. officinalis* extracts. Therefore, the anti-wrinkle activities of *A. officinalis* extracts were not related to their phenolic and flavonoid content. Furthermore, the anti-wrinkle activities were not related to their antioxidant activities. Although the antioxidant activity was higher in the tip extract than in the spear extract at 50 °C (Table 2), in contrast, the anti-wrinkle activity was lower in the tip extract than in the spear extract at 50 °C (Table 3).

Interestingly, the extract from *A. officinalis* spears dried at 50 °C for 24 h had the most potent anti-wrinkle activity since it possessed the (significantly) highest MMP-1, elastase, and hyaluronidase inhibition. This extract was more potent in MMP-1 inhibition than oleanolic acid and EGCG, the well-known natural MMP-1 inhibitors^27,28^. Furthermore, it was significantly more potent than gallic acid, quercetin, and rutin (*p* < 0.05). A likely explanation might be the synergistic effect of various bioactive components in the *A. officinalis* spear^29^. Additionally, it could be possible that *A. officinalis* extracts contain smaller molecular weight compounds with activities comparable to those of oleanolic acid (MW = 456.7 g/mol), EGCG (MW = 458.4 g/mol), gallic acid (MW = 170.1 g/mol), quercetin (MW = 302.2 g/mol), and rutin (MW = 610.5 g/mol). Therefore, the molar concentration of *A. officinalis* extracts might be higher than these standard compounds at the same concentration. These findings suggest the *A. officinalis* spear extract to be a promising potent anti-wrinkle ingredient for use in the cosmetic or cosmeceutical industries.

### Cytotoxicity effect of *A. officinalis* extracts on human PBMCs

The cytotoxic effect of *A. officinalis* extracts on human PBMCs, expressed as IC_20_ and IC_50_ values, are shown in Table 4. Although the results showed that the spear of *A. officinalis* was safer than the tip, the effective concentration of *A. officinalis* spears dried at 50 °C for 24 h (100 µg/mL), which showed the most potent anti-ageing activity, was less than the IC_20_ value (163.9 ± 26.3 µg/mL), i.e., more than 80% of human PBMCs were viable.

**Table 4:** Cytotoxicity effect of *A. officinalis* extracts on human PBMCs.

Sample	Drying temperature (°C)	Drying duration (h)	IC_20_ (μg/mL)	IC_50_ (μg/mL)
*A. officinalis* spear	50	32	> 200	> 200
	50	24	163.9 ± 26.3	> 200
	80	24	> 200	> 200
*A. officinalis* tip	50	24	122.4 ± 15.3	> 200
	80	24	125.7 ± 19.1	> 200

### Stability of *A. officinalis* extracts

**Figure 4:**
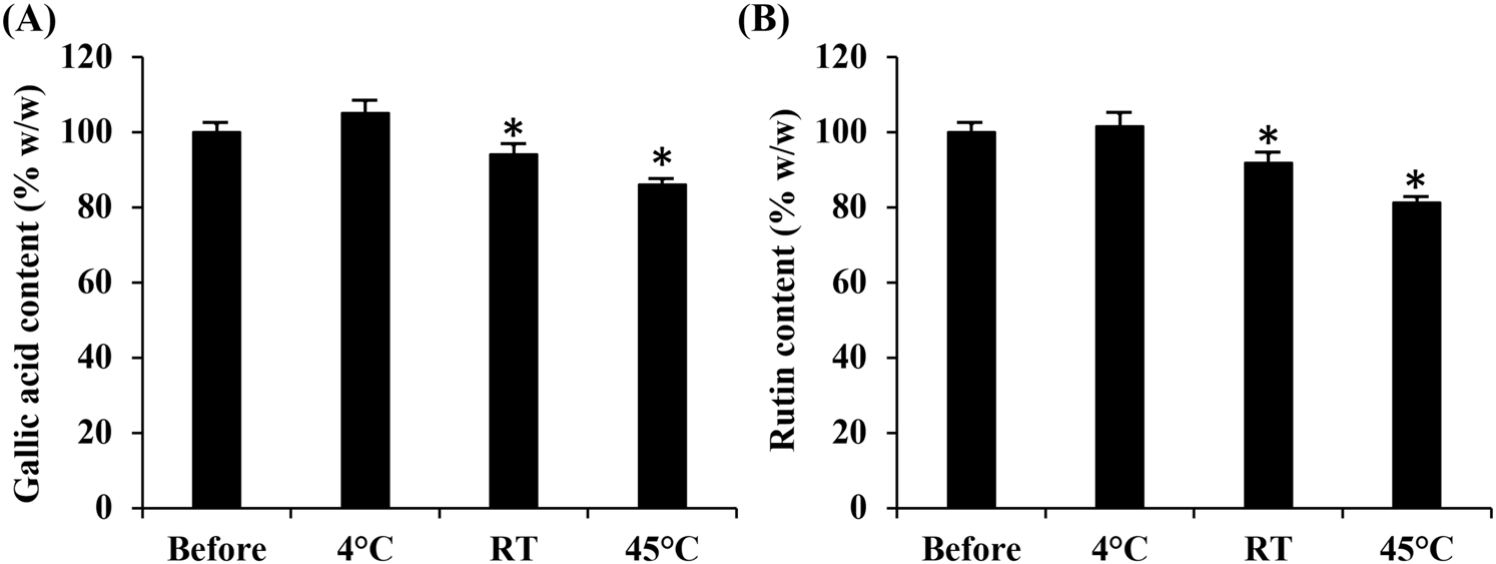
Gallic acid content (**A**) and rutin content (**B**) from the ethanolic extract of *A. officinalis* spears dried at 50 °C for 24 h before and after storage at various temperatures for 3 months. Asterisk (*) denotes significant difference between gallic acid and rutin content before and after the stability test (*p* < 0.05).

Since the ethanolic extract from *A. officinalis* spears dried at 50 °C for 24 h possessed the most potent inhibitory activities against MMP-1, elastase, and hyaluronidase, it was suggested for use as a natural anti-wrinkle agent. However, the temperature affected both the chemical composition and biological activity of *A. officinalis* ethanolic extracts. Therefore, the stability of selected *A. officinalis* spear extracts was investigated after storage at various temperatures for 3 months. The results as shown in Fig. 4 denote that the *A. officinalis* extract was stable after storage at low temperature (4 °C) since the gallic acid content (105 ± 3.5%) and rutin content (102 ± 3.4%) did not change from the initial level. However, degradation was detected after storage at room temperature and high temperature (45 °C), with the remaining gallic acid contents of 94 ± 0.4% and 86 ± 1.5%, respectively and the remaining rutin contents of 92 ± 2.7% and 81 ± 1.5%, respectively. Therefore, *A. officinalis* spear ethanolic extract should be kept at low temperature. Moreover, further developments of the nano delivery systems that could protect a compound from degradation, such as lipid nanoparticles, polymeric nanoparticles, vesicle systems, etc., are suggested.

## Materials and methods

### Plant material

The aerial part of CH105 *A. officinalis*, which was an in-house breed cultivated for contract farming of Cocos Agro Co., Ltd., Thailand, was collected from Sam Ngao District and Mae Sot District in Tak Province, in the North-western Thailand, during 2018 by the farm’s owner according to WHO Guidelines on Good Agricultural and Collection Practices (GACP) for Medicinal Plants^30^. The *A. officinalis* material was donated as a gift from Cocos Agro Co., Ltd., Tak, Thailand. Thereafter, *A. officinalis* plants were washed thoroughly using tap water and were dried at ambient temperature. The tip (the first 4 cm from the tip) and spear (below 4 cm from the tip) of *A. officinalis* were separated. The effects of different drying procedures were investigated. *A. officinalis* tips and spears were dried in a hot-air oven set at 50 °C or 80 °C for 24 h. Additionally, the spear part was dried in a hot-air oven set at 50 °C for 32 h. All dried *A. officinalis* materials were then ground into fine powder and kept in a sealed container at room temperature until further experiments.

### Chemical reagents

Rutin, quercetin, Trolox, gallic acid, Folin–Ciocalteu reagent, 2,2′-Azino-bis(3-ethylbenzothiazoline-6-sulfonic acid) (ABTS), collagenase from *Clostridium histolyticum* (ChC–EC.3.4.23.3), N-[3-(2-furyl) acryloyl]-Leu-Gly-Pro-Ala (FALGPA), elastase from porcine pancreatic (PE–E.C.3.4.21.36), N-Succinyl-Ala-Ala-Ala-p-nitroanilide (AAAPVN), hyaluronidase from bovine testis (E.C.3.2.1.3.5), sodium chloride (NaCl), calcium chloride (CaCl_2_), ferrous chloride (FeCl_2_), ferric chloride (FeCl_3_), 2,4,6 tripyridyl-s-triazine (TPTZ), glycerol, alcian blue 8GX, tricine, hyaluronic acid, sodium phosphate, and sodium acetate were purchased from Sigma-Aldrich (St. Louis, MO, USA). Fetal bovine serum (FBS) was purchased from GIBCO (Grand Island, NY, USA). Acetic acid, hydrochloric acid, and Tris base were purchased from Fisher Chem Alert (Fair Lawn, NJ, USA). Phosphoric acid and HPLC-grade acetonitrile were purchased from Merck (Darmstadt, Germany). Ethanol and dimethyl sulfoxide (DMSO) were analytical-grade and purchased from Labscan (Dublin, Ireland).

### Preparation of *A. officinalis* extract

The dried tip and spear powders of *A. officinalis* from different drying procedures were extracted by a maceration method. Briefly, 100 g of the *A. officinalis* dried powder was macerated in 500 mL of 95% ethanol for 3 days. After that, the resulting mixture was filtered through a Whatman no. 1 filter paper (GE Healthcare Bio-Sciences, Pittsburgh, PA, USA). The solvent was then removed under reduced pressure using a rotary evaporator (Tokyo Rikakikai, Co., Ltd., Tokyo, Japan) until dryness.

## Chemical composition determination

### Determination of rutin content using high-performance liquid chromatography (HPLC)

The chemical compositions of *A. officinalis* extracts were investigated using HPLC according to the previously described method with some modifications^31,32^. Briefly, gallic acid and rutin was used as a standard marker in the quantitative determination of *A. officinalis* extract. The HP1100 series HPLC was equipped with a UV/Vis spectrophotometric detector set at 210 nm (Hewlett Packard, Palo Alto, CA, USA). All samples and standard solutions were dissolved in HPLC-grade methanol and filtered through a 0.45 µm membrane filter (ANPEL Laboratory Technologies, Inc., Shanghai, China) before analysis. The injection volume of each sample was 20 µL. Chromatographic separation was performed on a Luna C18 column (5 µm, 4.6 × 250 mm) (Phenomenex, Torrance, CA, USA) using acetonitrile with 0.5% w/v phosphoric acid solution (A) and 0.5% w/v phosphoric acid aqueous solution (B) as a mobile phase, which was set at a flow rate of 1 mL/min. The gradient program was as follows: 0–2 min, 15–20% A; 2–8 min, 20–35% A; 8–10 min, 35–15% A; and 10–12, 15% A. All experiments were performed in triplicate.

### Determination of total phenolic content

The total phenolic contents of *A. officinalis* extracts were determined by the Folin–Ciocalteu method as previously described^33,34^. Briefly, 20 μL of the sample solution in DMSO at the concentration of 1 mg/mL was mixed with 180 μL of 1:10 diluted Folin-Ciocalteu reagent and incubated at room temperature for 4 min. Then, 80 μL of saturated sodium carbonate solution (0.7 M) was added and incubated at room temperature for another 2 h. The UV absorbance was measured at 750 nm using a multimode detector (Beckman Coulter DTX880, Fullerton, CA, USA). Gallic acid was used as a standard compound, and the total phenolic content is expressed as milligrams per gram of gallic acid equivalents (GAE). Three independent experiments repeated in triplicate were performed.

### Determination of total flavonoid content

The total flavonoid content of *A. officinalis* extracts was determined using an aluminum chloride colorimetric method as previously described^35–37^. Briefly, 100 µL of each sample solution (1 mg/mL) was added to 20 µL of 10% w/v aluminum chloride aqueous solution, 20 µL of 1.0 M potassium acetate solution, and 860 µL of deionized water, respectively. Thereafter, the resulting mixture was incubated at room temperature for 30 min in the dark. The presence of flavonoids was detected by spectrophotometric measurements using a 96-well microplate reader (BMG Labtech, Ortenberg, Germany) set at a wavelength of 415 nm. Quercetin was used as a standard compound, and the results are reported in terms of milligrams per gram of quercetin equivalent (QE). Three independent experiments repeated in triplicate were performed.

### Determination of antioxidant activities of *A. officinalis* extracts

#### 2,2-Azinobis (3-ethylbenzothiazoline-6-sulphonic acid) (ABTS) assay

The ABTS^+^ radical scavenging activity of *A. officinalis* extracts was investigated using an ABTS assay according to the method previously described^38,39^. Briefly, ABTS^+^ solution was prepared by mixing 7.0 mM ABTS^+^ and 2.45 mM potassium persulfate solution at a ratio of 2:3 and incubating at room temperature in the dark for 16 h. Then, 20 µL of each sample solution (1 mg/mL) was mixed with 180 µL of ABTS^•+^ solution. The presence of ABTS^•+^ was detected by spectrophotometric measurements using a 96-well microplate reader (BMG Labtech, Ortenberg, Germany) set at a wavelength of 750 nm after 5 min incubation. The results are reported in terms of Trolox equivalent antioxidant activity (TEAC). Ascorbic acid was used as a positive control in this experiment. Three independent experiments repeated in triplicate were performed.

#### Ferric reducing antioxidant power (FRAP) assay

Ferric reducing antioxidant power of *A. officinalis* extracts was investigated using an FRAP assay according to a method previously described^33,40^. Briefly, FRAP solution containing 0.3 M of acetate buffer (pH 3.6), 10 mM of TPTZ solution in 40 mM HCl, and 20 mM of FeCl_3_ at the ratio 10:1:1 was freshly prepared. Then, 20 µL of each sample solution (1 mg/mL) was added to 180 µL of FRAP solution and incubated at room temperature for 5 min. The presence of the colored Fe^2+^-TPTZ complex was detected by spectrophotometric measurements using a 96-well microplate reader (BMG Labtech, Ortenberg, Germany) set at the wavelength of 595 nm. The results are reported in terms of equivalent concentration (EC_1_), which represents the ferric reducing ability equivalent to 1 µM FeSO_4_. Ascorbic acid was used as a positive control. Three independent experiments repeated in triplicate were performed.

### Determination of anti-ageing activities of *A. officinalis* extracts

#### Matrix metalloproteinase-1 (MMP-1) inhibitory activity determination

The MMP-1 inhibitory activity of *A. officinalis* extracts was determined by spectrophotometric methods according to the previous studies^35,41^. Firstly, the enzyme activity of MMP-1 was determined before the experiment. Only enzyme activity of MMP-1 above 90% was used in further experiments. Briefly, the sample solution was added to 5 units/mL of MMP-1 solution and incubated for 15 min. Then, 2.0 M FALGPA in tricine buffer was added. The final concentration of *A. officinalis* extracts was 0.1 mg/mL. Immediately after the enzyme reaction, the absorbance of the resulting mixture was continuously measured for 20 min at a wavelength of 335 nm, using a multimode detector (BMG Labtech, Offenburg, Germany). Inhibitory activity against MMP-1 of each sample was calculated using the following equation: % inhibition = (1—*a*/*b*) × 100, where *a* is the reaction rate of the mixture with *A. officinalis* extracts, and *b* is the reaction rate of the mixture without *A. officinalis* extracts. Oleanolic acid and EGCG were used as positive controls in the present study. Three independent experiments repeated in triplicate were performed.

#### Elastase inhibitory activity determination

The elastase inhibitory activity of *A. officinalis* extracts was determined by spectrophotometric methods according to the previous studies^41,42^. Firstly, the enzyme activity of elastase was determined before the experiment. Only elastase enzyme activity above 90% was used in further experiments. Briefly, the sample solution was incubated with 4.5 unit/L of elastase for 15 min. Thereafter, 1.6 mM AAAVPN in tris HCI buffer (pH 8.0) was added. The final concentration of *A. officinalis* extracts was 0.1 mg/mL. Immediately after the reaction started, the absorbance of the resulting mixture was continuously measured for 20 min at a wavelength of 410 nm using a multimode detector (BMG Labtech, Offenburg, Germany). The inhibitory activity of each sample against elastase was calculated using the following equation: % inhibition = (1—*a*/*b*) × 100, where *a* is the reaction rate of the mixture with *A. officinalis* extracts, and *b* is the reaction rate of the mixture without *A. officinalis* extracts. EGCG was used as positive control in the present study. Three independent experiments repeated in triplicate were performed.

#### Hyaluronidase inhibitory activity determination

The inhibitory activity of *A. officinalis* extracts against hyaluronidase was determined by spectrophotometric methods according to the previous studies^41,42^. Firstly, the enzyme activity of hyaluronidase was determined before each experiment. Only enzyme activity of hyaluronidase above 90% was used in the further experiments. Briefly, the sample solution was incubated with 15 unit/mL hyaluronidase for 10 min in an incubator (BMG Labtech, Offenburg, Germany) at 37 °C. Thereafter, 0.03% w/v hyaluronic acid in phosphate buffer (pH 5.35) was added and incubated again in the same conditions for 45 min. The final concentration of *A. officinalis* extracts was 0.1 mg/mL. The precipitate of hyaluronic acid after addition of acid bovine serum albumin solution, composed of sodium acetate, acetic acid, and bovine serum albumin, was measured at 600 nm using a multimode detector (BMG Labtech, Offenburg, Germany). The inhibitory activity of each sample against hyaluronidase was calculated using the following equation: % inhibition = (1 − *a*/*b*) × 100, where *a* is the reaction rate of the mixture with *A. officinalis* extracts, and *b* is the reaction rate of the mixture without *A. officinalis* extracts. EGCG was used as positive control in the present study. Three independent experiments repeated in triplicate were performed.

### Cytotoxicity effect of *A. officinalis* extracts on peripheral blood mononuclear cells (PBMCs) by 3-(4,5-dimethylthiazol-2-yl)-2,5-diphenyl tetrazolium bromide (MTT) Assay

The cytotoxicity of *A. officinalis* extracts on PBMCs was evaluated using an MTT assay^43^. The PBMCs were collected from three healthy volunteers using the Ficoll–Hypaque density gradient centrifugation method. Then, PBMCs at 1.5 × 10^4^ cells/well were cultured and treated with various concentrations (0–200 μg/mL) of *A. officinalis* extracts and incubated for 48 h in a humidified 5% CO_2_ atmosphere MTT dye solution was added, and the plates were incubated at 37 °C for another 4 h. Then, the culture supernatant was removed, and 200 μL of DMSO was added to each well and mixed thoroughly to dissolve the formazan crystals. The absorbance was measured using a microplate reader (Metertech AccuReader M965) at 578 nm with a reference wavelength of 630 nm. High optical density readings corresponded to a high intensity of dye color, representing a high number of viable cells able to metabolize MTT salts. The percentage of cell viability was calculated by the following formula: % Cell viability = (*a*/*b*) × 100, where *a* is mean absorbance in the test well, and *b* is mean absorbance in the vehicle control well. Average cell survival obtained from triplicate determinations at each concentration was plotted as a dose–response curve. Three independent experiments were conducted. The 50% inhibitory concentration (IC_50_) of the active substances was determined as the lowest concentration that reduced cell growth by 50% in treated compared to untreated culture or vehicle control culture (0.2% DMSO in culture medium). The IC_50_ values were mean ± standard deviation (S.D.), and their activities were compared.

### Chemical stability of *A. officinalis* extracts

*A. officinalis* extracts were kept at various temperatures, including 4 °C, 30 °C, and 45 °C, for 3 months. Thereafter, the chemical composition of each *A. officinalis* extract was investigated using HPLC as previously described.

### Statistical analysis

The mean values of the results were calculated for all triplicate experiments, and the results are expressed as mean ± standard deviation. The data were subjected to a *t*-test and one-way ANOVA, with post-hoc Tukey test, and statistical significance was set at *p* < 0.05.
